# Low‐level gastrokine 2 promoted progress of NSCLC and as a potential biomarker

**DOI:** 10.1002/jcla.24213

**Published:** 2021-12-30

**Authors:** Xiang Yang, Wenjing Shi, Huixin Zhou, Xiaolou Huang, Lijuan Hu, Jiang Feng, Yumin Wang

**Affiliations:** ^1^ Department of Laboratory Medicine The First Affiliated Hospital of Wenzhou Medical University Wenzhou China

**Keywords:** biomarker, gastrokine 2, methylation, non‐small cell lung cancer

## Abstract

**Background:**

Gastrokine 2 (GKN2) is significantly downregulated in non‐small cell lung cancer (NSCLC) tissues than in normal tissues (NT), as assessed by mRNA microassay; however, the mechanism and clinical value of GKN2 is unknown in NSCLC.

**Methods:**

A total of 60 NSCLC samples and corresponding NT samples were prospectively collected GKN2 expression in NSCLC tissues was estimated. Also, the expression level of GKN2 promoter methylation and correlation with clinical data in NSCLC patients from public databases were analyzed. Cytology experiments were also carried out.

**Results:**

The GKN2 mRNA and protein expression level in NSCLC was significantly lower than that in the NT, and the GKN2 expression level in large tumors NSCLC was significantly lower than that in the small tumor group. Public data showed that expression of GKN2 in LUAD with P53 mutation group was lower than that of the P53 non‐mutation group, and GKN2 promoter methylation level of LUAD was significantly higher than its NT and close to age and clinical stage. Cell migration, invasion, and proliferation ability of GKN2 overexpressed were lower in A549 and PC9 groups than those in GKN2 overexpressed A549 and PC9 negative control groups, while the percentage of apoptotic cells increased in the GKN2 overexpressed A549 and PC9 groups. The DNMT3B mRNA expression levels were higher in PC9 and A549 cells than BEAS‐2B cells.

**Conclusion:**

The overexpression of GKN2 significantly inhibited cell proliferation, migration, and invasion and promoted apoptosis. Low‐level GKN2 promoted the progression of NSCLC via DNMT3B and is expected to be a biomarker for NSCLC.

## Graphical abstract

The expression of GKN2 mRNA in LUAD patients and cell lines showed low expression. Overexpression of GKN2 could significantly inhibit cell proliferation, migration, invasion, and apoptosis. Low‐level GKN2 promoted NSCLC progression by upregulating DNMT3B.

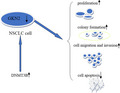

## 1 INTRODUCTION

According to data released by the International Agency for Research on Cancer, about 8 million people die from cancer each year. According to the 2015 China Cancer Statistics published in *CA Cancer J*. *Clin*. in January 2016, lung cancer is the leading cause of cancer deaths from malignant tumors in China. The incidence and mortality of tumors are highest in males.^1^ Despite continuous improvements in surgical resection, chemotherapy, and radiotherapy techniques, lung cancer is still prone to recurrence and fatality.^2^ Non‐small cell lung cancer (NSCLC) is the most common lung cancer, accounting for about 80% of the total number of lung cancers, including lung adenocarcinoma (LUAD) and lung squamous cell carcinoma (LUSC). Since the biological behavior of NSCLC is highly invasive and metastatic, about 80% of patients are at an advanced stage at the time of diagnosis, which often leads to treatment failure and death. Presently, the overall effect of NSCLC treatment is unsatisfactory, and the 5 years survival rate is only 10%–15%.^3^ In recent years, the incidence of NSCLC has been on the rise; however, its pathogenesis is not yet clarified, necessitating further exploration of NSCLC. Therefore, identifying specific molecular targets and early diagnostic biomarkers for NSCLC are the current hot spots in clinical research worldwide.

To further explore the genes of mRNAs associated with lung carcinogenesis, we used high‐throughput microarray technology to compare the differential mRNA expression profiles between NSCLC and its adjacent tissues and screen and identify a cohort of NSCLC‐associated mRNA molecules. Among these, the mRNA expression of Gastrokine 2 (GKN2) was significantly downregulated (fold‐change=−86.9873) among these candidate genes. This gene is located at 2p13.3 and has an mRNA length of 723 bp. Currently, no studies have reported GKN2 expression and lung cancer in China or abroad.

However, the literature reports on GKN2 and cancer‐related research mainly focused on gastric cancer. GKN includes three members, GKN1, GKN2, and GKN3. Among these, GKN2 was discovered as a member of GKN in 2002. It is not expressed or underexpressed in gastric cancer tissues and is closely related to the prognosis of gastric cancer patients. The overexpression of GKN2 inhibits the proliferation, migration, and invasion of gastric cancer cells through the interaction with GKN1, indicating GKN2 as a tumor suppressor.^4^, ^5^, ^6^ Currently, there is no report on the correlation between CNK2 and lung cancer.

In the present study, GKN2 expression levels were estimated by quantitative polymerase chain reaction (qPCR) in 60 pairs of NSCLC and normal lung tissue (NT) samples, and the correlation between GKN2 and clinical data from NSCLC patients was also analyzed. We also overexpressed GKN2 and observed the changes in the oncological behavior of A549 and PC9 cells.

## 2 MATERIALS AND METHODS

### 2.1 Patient samples

A total of 60 NSCLC samples and corresponding NT samples were prospectively collected from patients at the First Affiliated Hospital of Wenzhou Medical University, Wenzhou, China, from August 2018 to August 2021. The diagnosis of adenocarcinoma was confirmed by histopathology. The cohort comprised 31 females and 29 males, aged 24–80 years old. NSCLC and matched NT samples were snap‐frozen in liquid nitrogen immediately after resection according to the TNM clinical staging established by the American Joint Committee on Cancer (AJCC) and the Union for International Cancer Control (UICC) in 2002. Low‐expression groups were classified according to the expression level of GKN2. The study was approved by the ethics committee of the First Affiliated Hospital of Wenzhou Medical University (YS2018001), and all patients signed written informed consent for this study.

### 2.2 qPCR

Total RNA was extracted from frozen NSCLC tissues using TRIzol reagent (Invitrogen) and reverse transcribed into cDNA using RT Reagent Kit (Takara,) according to the manufacturer's instructions. Subsequently, the gene mRNA expression in NSCLC tissues was measured by qPCR using SYBR Premix Ex Taq on ABI 7000. qPCR was used to measure gene mRNA expression in NSCLC tissues. qPCR was used to measure the gene mRNA expression in NSCLC tissues. The detailed primer sequences for qPCR are shown in Table 1. An equivalent of 2 mg total RNA was transcribed into cDNA. The 20 μl PCR reaction consisted of 10 μl SYBR Premix (2X), 2 μl cDNA template, 1 μl PCR forward primer (10 mM), 1 μl PCR reverse primer (10 mM), and 6 μl double‐distilled water. The qPCR reaction consists of an initial denaturation step at 95°C for 10 min; 40 cycles of 95°C for 5 s and 60°C for 30 s; final extension at 72°C for 5 min. All experiments were performed in triplicate, and all samples were normalized to β‐actin. The median of each triplicate was used to calculate the relative mRNA concentration (△Ct = Ct median lncRNA −Ct median β‐actin), and 2^−△△Ct^ was calculated.^7^

**TABLE 1:** Primer sequences for qRT‐PCR

Gene	Primer sequences PCR product(bp)	
	Forward	Reverse
GKN2	ACGTGGATTGGTTCCTGCTT	CCAGCACCGACATTATGTGT 150
β‐actin	CCTGGCACCCAGCACAAT	GCTGATCCACATCTGCTGGAA 158
DNMT1	GCGGCTCAAAGATTTGGAAAGA	CCAGGTAGCCCTCCTCGGAT 161
DNMT3B	GTCGTGCAGGCAGTAGGAAAT	GAAGCCATTTGTTCTCGGCT 178
DNMT3A	CGCGATTTCTCGAGTCCAAC	TTGGCTATCCTGCCATGCTC 169

### 2.3 Biochemical analysis

A 5 ml volume of patients’ fasting blood was collected, and the serum was separated by centrifugation at 3500 rpm for 5 min and stored at −80℃. The levels of carcinoembryonic antigen (CEA), neuron‐specific enolase (NSE), and cytokeratin 19 fragment (CYFRA21‐1) in patient serum were measured by electrochemiluminescence. The reagents, quality control materials, and calibration solutions were obtained from Roche (Germany).

### 2.4 Cell culture

Normal human bronchial epithelial cells BEAS‐2B and three independent NSCLC cell lines (A549 and PC9) were purchased from the cell bank of the Chinese Academy of Sciences and cultured at 37°C in complete medium (containing 10% fetal serum and 90% RPMI1640) at 5% CO_2_. The complete medium was changed at least every two days.

### 2.5 Transfection

The overexpression plasmid pcDNA‐GKN2 and the control vector pcDNA‐3.1 were purchased from Suzhou Imax Biotechnology. Lipofectamine 3000 (Invitrogen) was used for cell transfection.

### 2.6 Cell viability assay

Cell viability was assessed using the Cell Counting Kit 8 (CCK‐8, Corning Corporation,) according to the manufacturer's protocol. Briefly, 3000 cells were inoculated into 96‐well plates supplemented with 10% FBS. The following day, GKN2‐overexpressed cells were incubated with CCK8 for 1 h, and absorbance was measured at 450 nm on days 1, 3, 5, and 7 using a multifunctional enzyme marker (Tecan). The experiments were performed in quadruplicate.

### 2.7 Cell migration and invasion assays

Migration and invasion assays were performed in 24‐well plates (Millipore) with 8.0 μm well inserts. For migration assays, 2 × 10^4^ cells were seeded in the upper compartment of the transwell insert. Invasion assays were performed with a matrix gel‐coated filter (Sigma Corporation,). The cells were incubated for 24 and 48 h, respectively, fixed with methanol and stained with 0.1% (w/v) crystal violet, followed by bleaching with 33% acetic acid. The absorbance was measured at 570 nm. Each experiment was performed in triplicate.

### 2.8 Flow cytometry to detect apoptosis

Apoptosis Detection Kit (KeyGEN,) was used for apoptosis detection. After washing the cells twice with phosphate‐buffered saline (PBS), cells were resuspended in 500 μl Binding Buffer, 5 μl Annexin V‐APC, and 5 μl 7‐AAD dye. Flow cytometry was performed for 5–15 min in the dark (Beckman).

### 2.9 Public database analyzes the expression level of GKN2 protein and promoter methylation in NSCLC patient tissues and correlation with clinical data

GNK2 mRNA and protein expression levels were analyzed in NSCLC from the GEPIA database (http://gepia.cancer‐pku.cn/index.html). According to http://ualcan.path.uab.edu, we analyzed the GNK2 promoter methylation levels in LUAD and its correlation with clinical data.

### 2.10 Statistical methods

The two groups were compared using Student's *t*‐test, and enumeration data were analyzed using chi‐square test; *p* < 0.05 was statistically significant.

## 3 RESULTS

### 3.1 Expression level of GKN2 in lung cancer and adjacent tissues and correlation with clinical data

**FIGURE 1:**
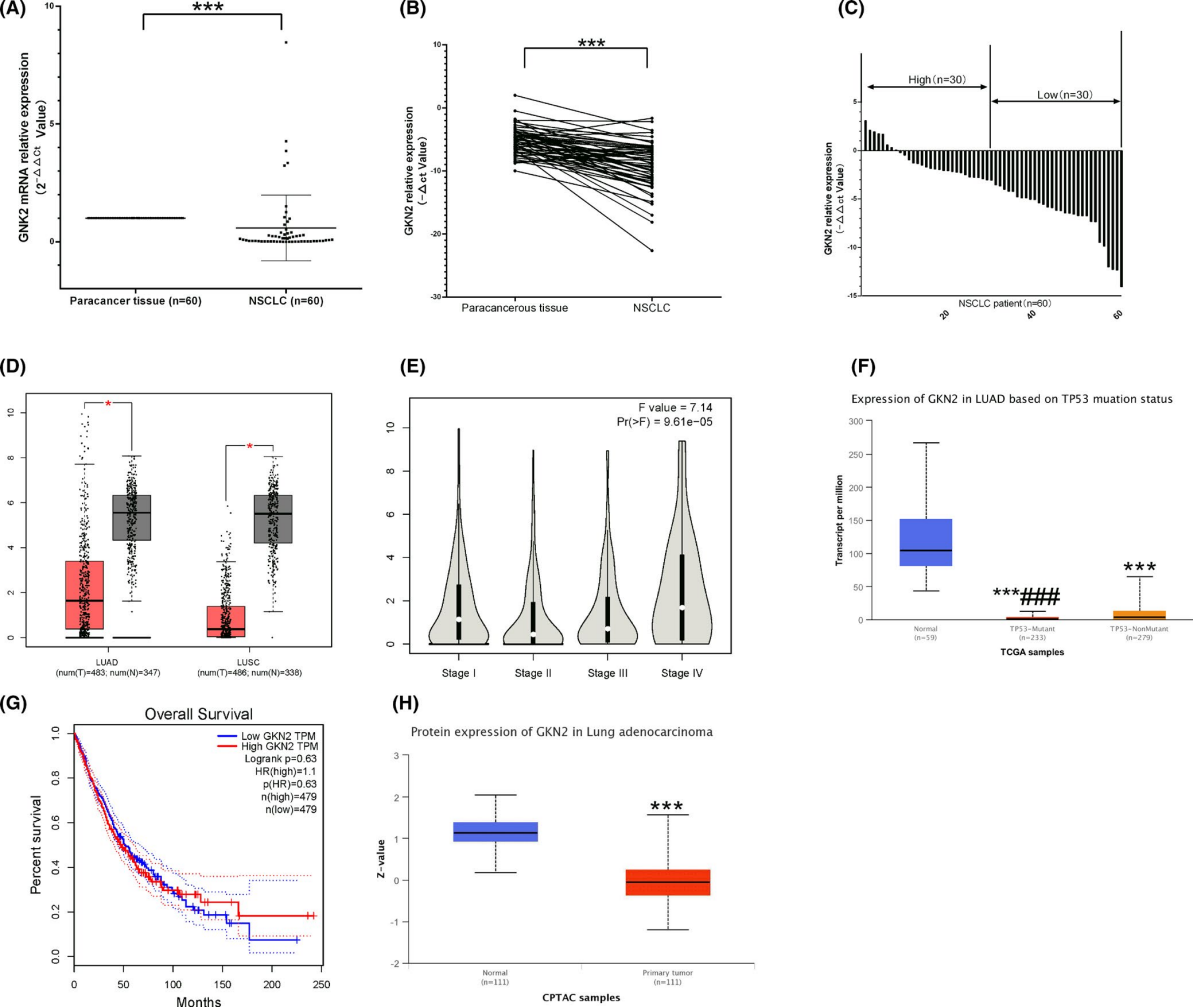
Expression level of GKN2 in lung cancer and adjacent tissues. (A) GKN2 expression level of NSCLC was significantly lower than its NT (*p* < 0.001) in the scatter graph. (B) GKN2 expression level was significantly lower in NSCLC than its NT (*p* < 0.001) in the line graph. (C) GKN2 expression level was significantly lower in NSCLC than in NT (*p* < 0.001) in the bar graph. (D) Analysis of GEPIA data (http://gepia.cancer‐pku.cn/index.html) showed significantly lower GNK2 in both LUAD and LUSC than the adjacent tissues. (E) GNK2 levels in LUAD and LUSC were associated with clinical staging (*p* = 9.61e‐05). (F) Further analysis showed that GNK2 expression in LUAD was lower in the P53 mutant group than in the P53 non‐mutant group (*p* < 0.001). (G) Survival analysis did not show any statistical difference in the survival time between high and low expression of GKN2 in NSCLC patients (Log‐rank *p* = 0.63, http://gepia.cancer‐pku.cn/detail.php?gene=GKN2). (H) GNK2 protein expression levels were significantly lower in NSCLC than the adjacent cancer tissues (http://ualcan.path.uab.edu/cgi‐bin/CPTAC‐Result.pl?genenam=GKN2&ctype=LUAD, *p* < 0.001). Note: Z‐values represent standard deviations from the median across samples for the given cancer type. ***p < 0.001; ###*p* < 0.001

According to Figure 1A,B,C, the GKN2 mRNA expression level in NSCLC was significantly lower than in NT (*p* < 0.001). We found that the GKN2 levels in large tumors (>2 cm) of NSCLC were significantly lower than those in the small tumors (≤2 cm) (*p* < 0.001). No difference was detected in GKN2 levels with respect to clinical parameters, such as age, gender, lymph node metastasis, TNM stage, CEA, NSE, and CYFRA21‐1 (*p* > 0.999, *p* = 0.438, *p* > 0.999, *p* = 0.299, *p* = 0.607, *p* = 0.229, and *p* = 0.700; Table 2).

**TABLE 2:** Correlation between the expression level of GKN2 and the clinical characteristics of NSCLC

Parameter	N(60)	Relative GNK2 expression		
		Low(30)	High(30)	*p* value
Age/years				>0.999
≤60	24	12	12	
>60	36	18	18	
Gender				0.438
Male	29	13	16	
Female	31	17	14	
Tumor size				<0.001
≤2 cm	37	12	25	
>2 cm	23	18	5	
Lymph node metastasis				>0.999
Negative	53	26	27	
Positive	7	4	3	
TNM stage				0.299
Ⅰ	50	23	27	
Ⅱ‐Ⅳ	10	7	3	
CEA (ng/ml)				0.607
≤5	46	23	23	
>5	14	7	7	
CY211(ng/ml)				0.229
≤3.3	36	20	16	
>3.3	24	10	14	
NSE (ng/ml)				0.700
≤15	36	18	18	
>15	24	12	12	

The expression of GKN2 in NSCLC was analyzed from the GEPIA database (http://gepia.cancer‐pku.cn/index.html), and the results showed that the level of GKN2 expression in NSCLC was significantly lower than that of adjacent tissues in LUAD and LUSC (Figure 1D) and was related to the clinical stage (*p* = 9.61e‐05) (Figure 1E). Further analysis showed that the expression of GKN2 in LUAD with P53 mutation group was lower than that of the P53 non‐mutation group (Figure 1F *p* < 0.001), and the prognosis of NSCLC did not differ significantly from the high or low level of GKN2 mRNA expression (*p* = 0.63) (Figure 1G). Similarly, the GKN2 protein expression level of NSCLC was significantly lower than its adjacent cancer tissues (http://ualcan.path.uab.edu/cgi‐bin/CPTAC‐Result.pl?genenam=GKN2&ctype=LUAD) (*p* < 0.001, Figure 1H).

### 3.2 Promoter methylation of GKN2 in LUAD was higher than adjacent tissues and the correlation with clinical stage and age

**FIGURE 2:**
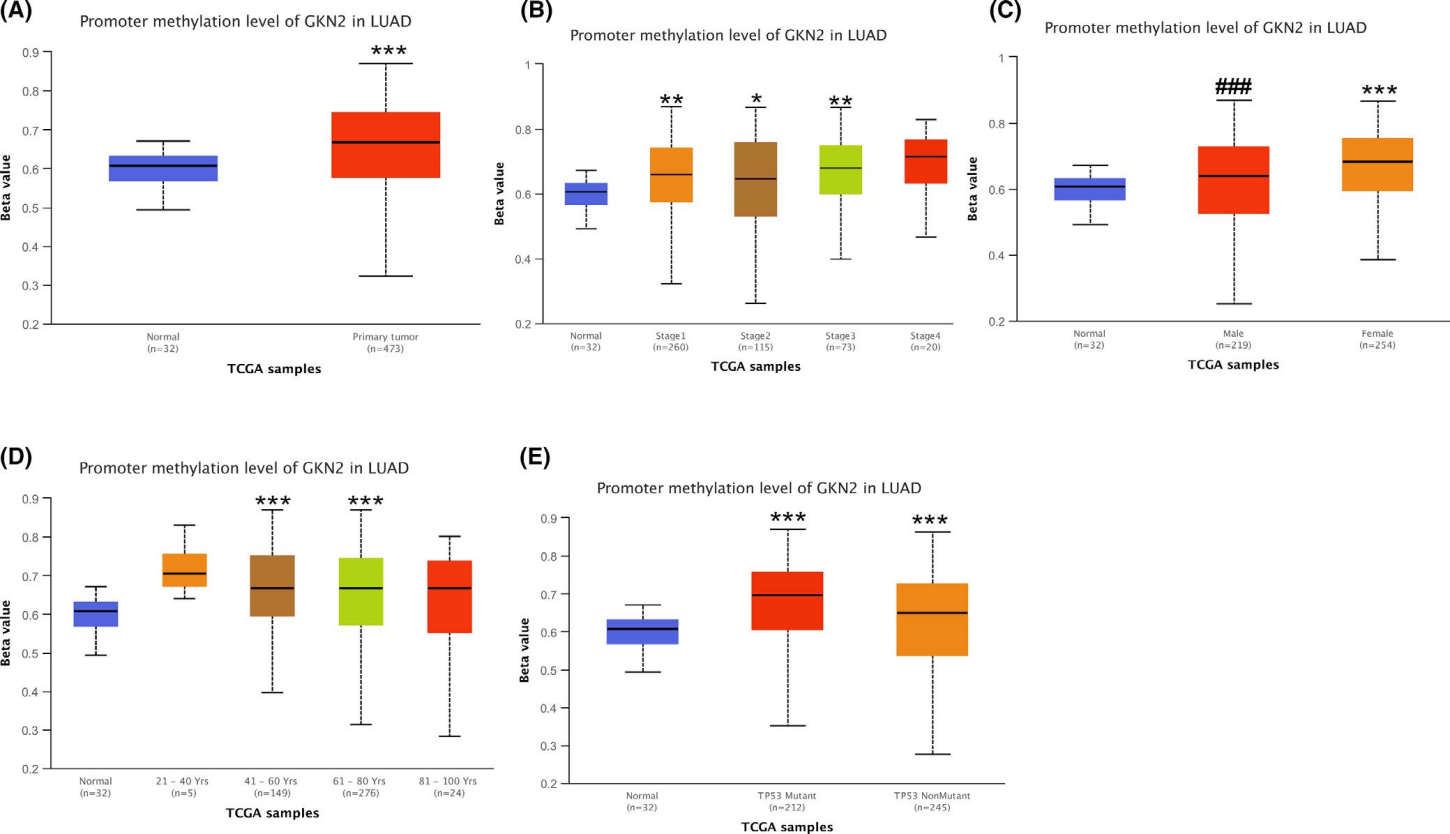
Analysis of GNK2 promoter methylation in lung cancer and paraneoplastic tissues and the correlation with clinical data. (A) According to http://ualcan.path.uab.edu, the level of GNK2 promoter methylation was significantly higher in LUAD than in the adjacent cancer tissues (*p* < 0.001). (B) Further analysis showed that GNK2 promoter methylation levels were higher in stage I, II, and III tumors than in the adjacent cancer tissues (*p* < 0.01, *p* < 0.05, *p* < 0.01), while no significant difference was observed in stage IV LUAD. (C) GNK2 promoter methylation levels were higher in men and women with LUAD than in the adjacent cancer tissues (*p* < 0.001 and *p* < 0.001). (D) GNK2 promoter methylation levels were significantly higher in 41–60‐ and 61–80‐year‐old groups than in the paracancerous tissues (*p* < 0.001 and *p* < 0.001), while no statistically significant difference was detected between 21–40‐ and 81–100‐year‐old groups and paracancerous tissues. (E) The level of GNK2 promoter methylation was significantly higher in both P53 mutation and P53 non‐mutation groups in LUAD patients than in their paraneoplastic tissues (*p* < 0.001 and *p* < 0.001). Note: β‐value indicates the level of DNA methylation ranging from 0 (unmethylated) to 1 (fully methylated). *p < 0.05, **p < 0.01, ***p < 0.001; ###*p* < 0.001

According to http://ualcan.path.uab.edu/cgi‐bin/TCGA‐methyl‐Result.pl?genenam=GKN2&ctype=LUAD, GKN2 promoter methylation level of LUAD was significantly higher than its adjacent cancer tissues (Figure 2A *p* < 0.001). Further analysis showed that GKN2 promoter methylation level of stage I, stage II, and stage III was significantly higher than the adjacent cancer tissues (Figure 2B; *p* < 0.01, *p* < 0.05, *p* < 0.01) while stage IV did not show any significant statistical difference in LUAD. GKN2 promoter methylation levels in males and females from LUAD patients were higher than the adjacent cancer tissues (*p* < 0.001, *p* < 0.001; Figure 2C). GKN2 promoter methylation level of 41–60‐ and 61–80‐year‐old group was significantly higher than its adjacent cancer tissues (*p* < 0.001, *p* < 0.001) while 21–40‐ and 81–100‐year‐old group did not differ significantly in LUAD (Figure 2D). GKN2 promoter methylation level in P53 mutation and P53 non‐mutation groups from LUAD patients was higher than the adjacent cancer tissues (*p* < 0.001, *p* < 0.001; Figure 2E).

### 3.3 Construction of A549 and PC9 GKN2 overexpression (OE) groups and GKN2 reduced the proliferation in NSCLC

**FIGURE 3:**
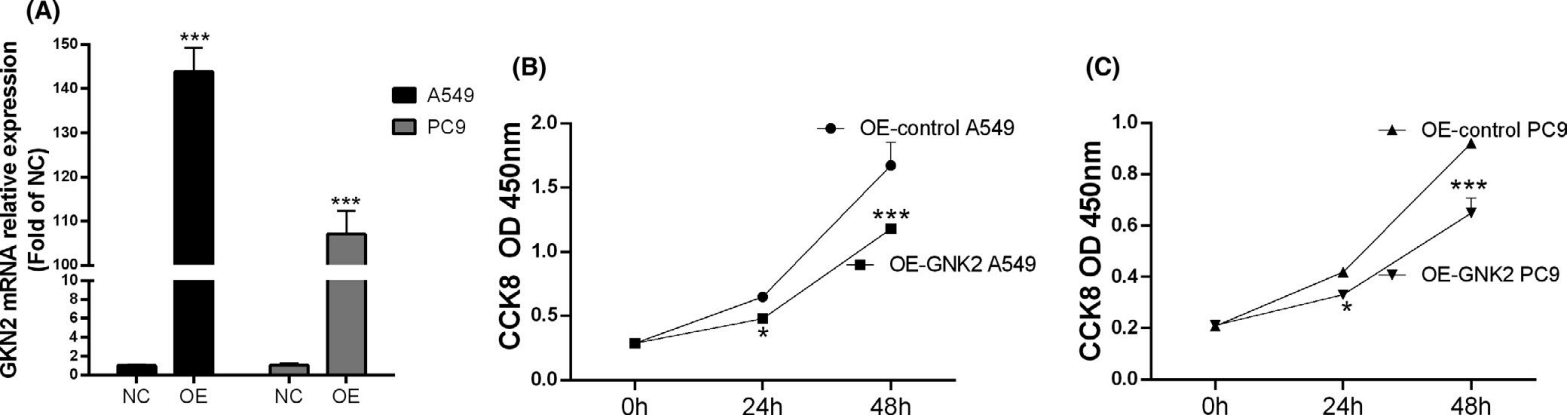
A549 and PC9 GKN2 OE groups were constructed and GKN2‐reduced NSCLC proliferation. (A) We selected PC9 and A549 cells for cotransfection with the GKN2 overexpression plasmid. Post‐transfection qPCR data showed that the expression levels of the A549 GKN2 OE and PC9 GKN2 OE groups were significantly higher than those of the A549 GKN2 OE NC and PC9 GKN2 OE NC groups. (B) OD450 of the A549 and PC9 groups increased gradually compared to the GKN2 OE A549 and PC9 NC groups. (C) OD450 was significantly decreased at 24 h and 48 h compared to 0 h (*p* < 0.05, *p* < 0.001). *** *p* < 0.001

PC9 and A549 cells were cotransfected with the GKN2 overexpressed plasmid. The qPCR results after transfection showed that the expression levels in the A549 GKN2 OE and PC9 GKN2 OE groups were significantly higher than those in the A549 GKN2 OE NC and PC9 GKN2 OE NC groups (Figure 3A). This indicated successful construction of A549 GKN2 OE and PC9 GKN2 OE groups. Figure 3B,3C shows that OD450 nm of different A549 and PC9 groups increased in a time‐dependent manner compared to the GKN2 OE A549 and PC9 NC groups. The absorbance at OD450 nm was significantly decreased at 24 h and 48 h compared to 0 h (*p* < 0.05 and *p* < 0.001, respectively), indicating that the cell proliferation ability of A549 and PC9 was decreased significantly after GKN2 overexpression.

### 3.4 GKN2 inhibited cell migration and invasion in NSCLC

**FIGURE 4:**
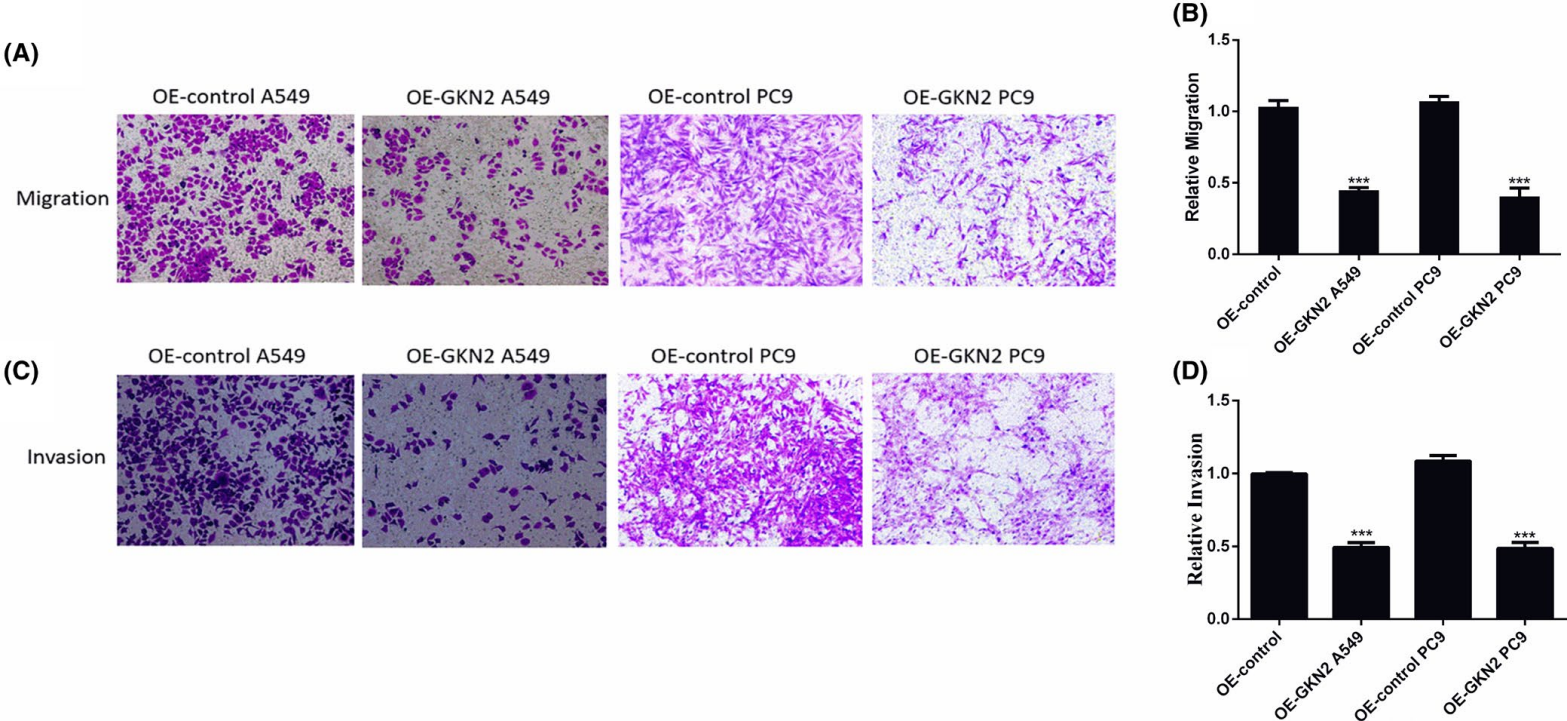
GKN2 inhibited cell migration and invasion in NSCLC. (A‐B) Cell migration showed lower cell numbers in GKN2 OE A549 and PC9 than in GKN2 OE A549 and PC9 NC groups (*p* < 0.001, *p* < 0.001). (C‐D) The cell number of GKN2 OE A549 and PC9 was lower than that of GKN2 OE A549 and PC9 NC groups (*p* < 0.01, *p* < 0.01), suggesting that GKN2 inhibited the cell invasion ability of NSCLC. **p < 0.01, ***p < 0.001

Cell migration showed lower cell numbers in GKN2 OE A549 and PC9 than in GKN2 OE A549 and PC9 NC groups (*p* < 0.001 and *p* < 0.001, respectively; Figure 4A,B); therefore, GKN2 inhibited cell migration in NSCLC. According to Figure 4C and D, the cell number of GKN2 OE A549 and PC9 was lower than that of GKN2 OE A549 and PC9 NC groups (*p* < 0.01 and *p* < 0.01, respectively), suggesting that GKN2 inhibited the cell invasion ability of NSCLC.

### 3.5 GKN2 expression level was associated with cell apoptosis in NSCLC

**FIGURE 5:**
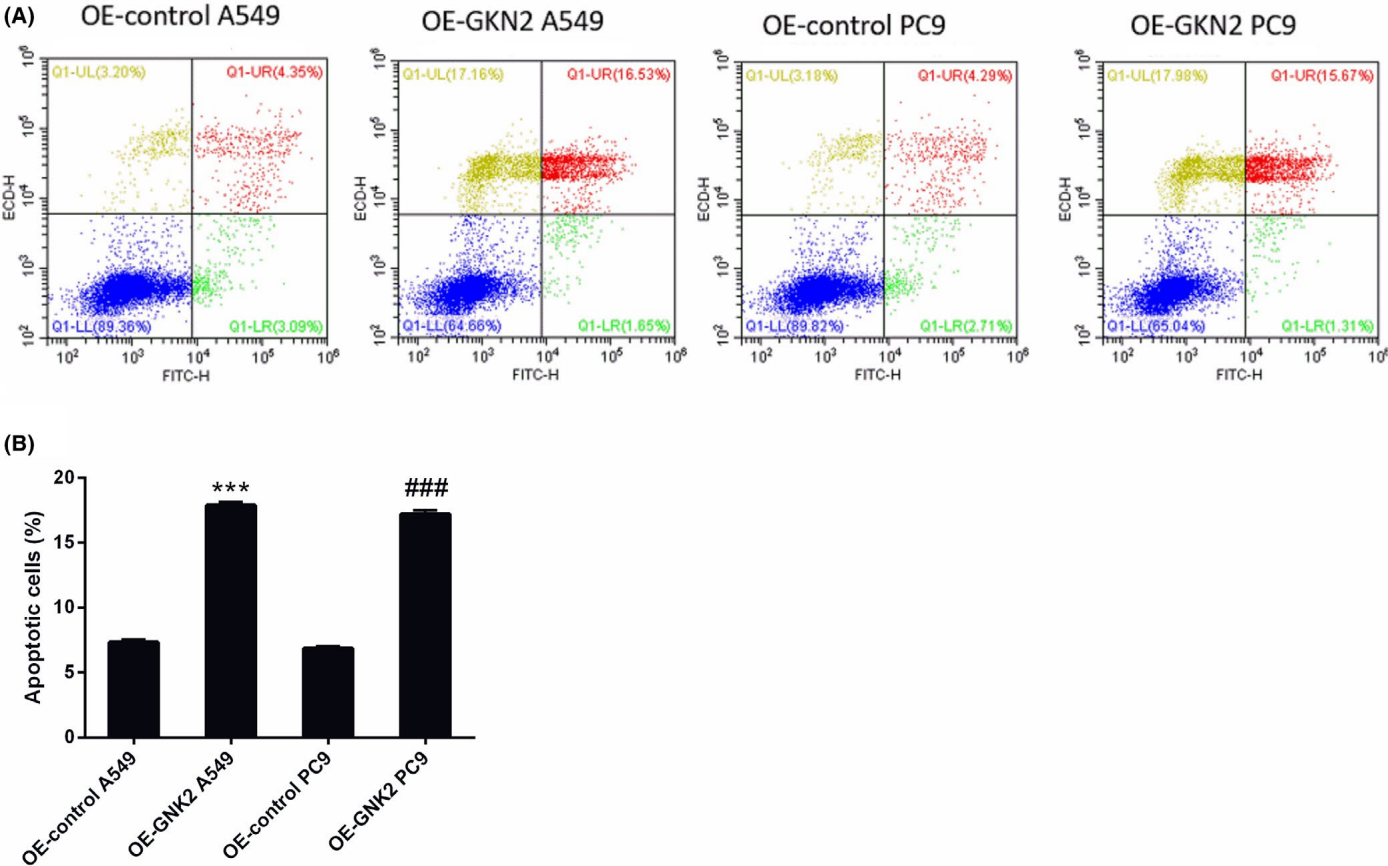
GKN2 expression level was associated with cell apoptosis in NSCLC. (A) Flowchart of apoptosis of GKN2 OE A549 NC group, GKN2 OE A549 group, GKN2 OE PC9 NC group, and GKN2 OE PC9 group. (B) The percentage of apoptotic cells was significantly increased in the GKN2 OE A549 (17.43 ± 1.12%, *p* < 0.001) and PC9 (16.91 ± 1.02%, *p* < 0.001) NC groups compared to the GKN2 OE A549 (7.31 ± 0.11%) and PC9 (6.94 ± 0.20%) NC groups. ***p < 0.001, ###*p* < 0.001

Figure 5A shows the apoptosis maps of the 4 groups of cell lines. Furtherly analysis hinted that the percentage of apoptotic cells was significantly increased in the GKN2 OE A549 (17.43 ± 1.12%, *p* < 0.001) and PC9 (16.91 ± 1.02%, *p* < 0.001) NC groups compared to the GKN2 OE A549 (7.31 ± 0.001) NC group and PC9 (6.94 ± 0.20%) NC groups, seen in Figure 5B.

### 3.6 DNMT3B might be involved in the methylation of NSCLC with low expression of GKN2

**FIGURE 6:**
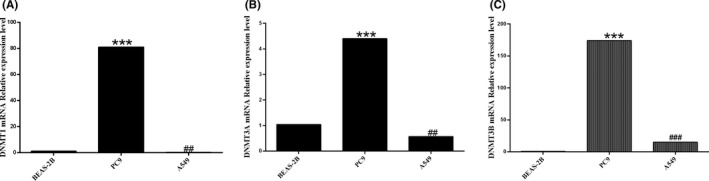
Expression level of DNA methylase mRNA in NSCLC cell line. We found that the DNMT1 mRNA expression level of PC9 was higher than that of BEAS‐2B (*p* < 0.001), while the expression level of A549 was lower than that of BEAS‐2B (*p* < 0.01). Compared to BEAS‐2B, DNMT3A mRNA expression level was increased in PC9 (*p* < 0.001), while DNMT3A mRNA expression level was decreased in the A549 cell line (*p* < 0.01). DNMT3B mRNA expression levels were higher in PC9 and A549 cell lines than in BEAS‐2B (*p* < 0.001, *p* < 0.001). **p < 0.01, ***p < 0.001; ##*p* < 0.01, ###*p* < 0.001

Figure 6 shows that the DNMT1 mRNA expression was higher than that of BEAS‐2B in PC9 (*p* < 0.001), while that in A549 was lower compared to BEAS‐2B (*p* < 0.01). Compared to BEAS‐2B, DNMT3A mRNA expression was increased in PC9 (*p* < 0.001) but was decreased in the A549 cell line (*p* < 0.01). Moreover, DNMT3B mRNA expression levels were higher in PC9 and A549 cell lines than BEAS‐2B (*p* < 0.001 and *p* < 0.001, respectively). Combining the results in Figures 1 and 2, we speculated that DNMT3B plays a critical role in the occurrence of GKN2 methylation.

## 4 DISCUSSION

Based on the study by Dai et al., GKN2 was decreased or lacked expression in gastric cancer cell lines, BGC‐823, SGC‐7901, and AGS.^8^ Baus‐Loncar et al. demonstrated a critical role of GKN2 in maintaining gastric mucosal homeostasis by regulating the activity of GKN1.^9^

In this study, we found that the expression level of GKN2 in NSCLC was significantly lower than its adjacent cancer tissues, and the expression in large tumor (>2 cm) NSCLC was significantly lower than that in small tumors (≤2 cm). This suggested that GKN2 is lowly expressed in NSCLC tissues and cell lines, and we confirmed that it was lowly expressed by TCGA data. This phenomenon is correlated with clinical staging. Similarly, the GKN2 protein level was significantly lower in NSCLC than in the adjacent cancer tissues. Further cytological experiments confirmed that cell migration, invasion, and proliferation of A549 and PC9 cells were significantly decreased after GKN2 overexpression, while apoptosis was significantly enhanced.

According to http://ualcan.path.uab.edu, the GKN2 promoter methylation level of LUAD was significantly higher than the adjacent cancer tissues. Further analysis showed that GKN2 promoter methylation level of stage I, II, and III was significantly higher than the adjacent cancer tissues, indicating GKN2 promoter hypermethylation in NSCLC. RAD9 was epigenetically regulated by DNMT1 and DNMT3B, and subsequent RAD9 overproduction promotes prostate tumorigenesis by targeting hypermethylation.^10^ The methylation profile of the CDKN2B promoter, a cell cycle inhibitor gene, was reduced in cells overexpressing miR‐29b that inhibits bile duct cancer progression by releasing DNMT3B‐mediated inhibition of CDKN2B expression.^11^ FOXC1 induces CTH promoter DNA hypermethylation through upregulation of DNMT3B to promote proliferation and metastasis in primary hepatocellular carcinoma.^12^ Next, we found that DNMT1 mRNA expression levels are higher in PC9 than BEAS‐2B, while DNMT1 expression was lower in A459 than BEAS‐2B cells. Moreover, DNMT3A mRNA expression levels were elevated in PC9 compared to BEAS‐2B but reduced in A549 cell lines. DNMT3B mRNA expression levels were higher in PC9 and A549 cell lines than BEAS‐2B. Thus, we speculated that DNMT3B might play a significant role in the occurrence of GKN2 methylation.

In summary, GKN2 mRNA expression was downregulated in NSCLC patients and cell lines, and GKN2 promoter hypermethylation was detected in NSCLC. The overexpression of GKN2 significantly inhibited cell proliferation, migration, invasion, and apoptosis. Thus, it seems a promising biomarker for NSCLC.

## CONFLICT OF INTERESTS

The authors declare that they have no competing interests.

## Data Availability

The corresponding author should be contacted for any data.
